# Extensive Independent Amplification of Platy-1 Retroposons in Tamarins, Genus *Saguinus*

**DOI:** 10.3390/genes14071436

**Published:** 2023-07-13

**Authors:** Jessica M. Storer, Jerilyn A. Walker, Thomas O. Beckstrom, Mark A. Batzer

**Affiliations:** 1 Department of Biological Sciences, Louisiana State University, 202 Life Sciences Building, Baton Rouge, LA 70803, USA; 2 Institute for Systems Biology, Seattle, WA 98109, USA; 3 Department of Oral and Maxillofacial Surgery, University of Washington, 1959 NE Pacific Street, Health Sciences Building B-241, Seattle, WA 98195, USA

**Keywords:** Callithrichidae, tamarin, Platy-1, *Saguinus*, Platyrrhine SINE, MEI

## Abstract

Platy-1 retroposons are short interspersed elements (SINEs) unique to platyrrhine primates. Discovered in the common marmoset (*Callithrix jacchus*) genome in 2016, these 100 bp mobile element insertions (MEIs) appeared to be novel drivers of platyrrhine evolution, with over 2200 full-length members across 62 different subfamilies, and strong evidence of ongoing proliferation in *C. jacchus*. Subsequent characterization of Platy-1 elements in *Aotus*, *Saimiri* and *Cebus* genera, suggested that the widespread mobilization detected in marmoset (family Callithrichidae) was perhaps an anomaly. Two additional Callithrichidae genomes are now available, a scaffold level genome assembly for *Saguinus imperator* (tamarin; SagImp_v1) and a chromosome-level assembly for *Saguinus midas* (Midas tamarin; ASM2_v1). Here, we report that each tamarin genome contains over 11,000 full-length Platy-1 insertions, about 1150 are shared by both *Saguinus* tamarins, 7511 are unique to *S. imperator*, and another 8187 are unique to *S. midas*. Roughly 325 are shared among the three callithrichids. We identified six new Platy-1 subfamilies derived from Platy-1-8, with the youngest new subfamily, Platy-1-8c_*Saguinus*, being the primary source of the *Saguinus* amplification burst. This constitutes the largest expansion of Platy-1 MEIs reported to date and the most extensive independent SINE amplification between two closely related species.

## 1. Introduction

Platyrrhine specific Platy-1 retroposons were first discovered in the genome of the common marmoset (*C. jacchus*) [1], a member of the Callithrichidae family and the first platyrrhine primate to have whole genome sequencing (WGS) [2]. Platy-1 elements are a type of SINE (Short INterspersed Element), are approximately 100 bp long and ending in a 3′ A-rich tail. Platy-1 SINEs are similar to the primate specific *Alu* SINE in that they are non-autonomous and mobilize via a “copy and paste” mechanism through an RNA intermediate, utilizing the enzymatic machinery of autonomous LINE (L1) elements, a process termed “target-primed reverse transcription” (TPRT) [3,4]. Platy-1 elements possess A- and B-box internal control regions as illustrated previously [1], further indication of transcription by Pol III and genomic integration via TPRT. Roughly 2200 Platy-1 elements, characterized into 62 subfamilies, were ascertained from *C. jacchus* [calJac3] in that initial study, and their amplification dynamics ranged from a slow, mostly linear propagation during early Platy-1 evolution in all platyrrhines, to a star-like burst of younger subfamilies unique to the common marmoset [1]. As WGS became available for other platyrrhine genomes, Platy-1 elements were characterized in squirrel monkey (*Saimiri boliviensis;* saiBol1) (Cebidae), capuchin monkey (*Cebus imitator*; Cebus_imitator-1.0) (Cebidae) and owl monkey (*Aotus nancymaae*; Anan_1.0) (Aotidae) [5]. Platy-1 elements have reportedly been nearly quiescent in the *Saimiri* and *Cebus* lineages for millions of years, whereas Platy-1 mobilization in owl monkey (*Aotus*) appears to have resumed with recent discernible activity leading to the discovery of two new subfamilies, Platy-1-4b_*Aotus* and 4b3_*Aotus*. However, all three genera (*Saimiri*, *Cebus* and *Aotus*) contain only a fraction of the number of Platy-1 elements compared to marmoset [5].

Recently, WGS became available for two additional members of the Callithrichidae family, a scaffold level genome assembly for *S. imperator* (tamarin; SagImp_v1) and a chromosome-level assembly for *S. midas* (Midas tamarin; ASM2_v1). Recent studies have proposed that phenotypic differences among tamarin species warrant the separation of genus *Saguinus* into three subgenera, *Leontocebus*, *Saguinus* and *Tamarinus* [6]. Recently, Brcko et al. (2022) [7] recommended elevating these subgenera to full genera, as well as adding genus *Oedipomidas*, providing the oedipus group of tamarins a separate genus. Under this proposal, *S. imperator* would become *Tamarinus imperator*, while *S. midas* would remain in genus *Saguinus*. These taxonomic reclassifications may become widely adopted in future studies; however, in this study we have retained the conventional *S. imperator* and *S. midas* designations to be consistent with the names of the genome assemblies used here. 

The purpose of this study was to computationally ascertain full-length Platy-1 elements from both newly assembled tamarin genomes and to assess the amplification dynamics and subfamily structure as compared to previous Platy-1 analyses. The data reported in this study suggest that *Saguinus* tamarins are experiencing rapid independent expansion of Platy-1 MEIs, far surpassing that previously reported for the marmoset [1].

## 2. Materials and Methods

### 2.1. Full-Length Platy-1 Elements

The scaffold level genome assembly for *S. imperator* [SagImp_v1] (GCA_004024885.1 _SagImp_v1_BIUU), the chromosome-level genome assembly for *S. midas* [ASM2_v1] (GCA_021498475.1_ASM2149847v1), and a more recent genome assembly for *C. jacchus* [calJac4] (GCA_009663435.2_Callithrix_jacchus_cj1700_1.1 [calJac4] were obtained from the National Center for Biotechnology Information. Each genome was subjected to RepeatMasker [8] (RepeatMasker-Open-4.0; accessed on 14 June 2023) analysis using a custom library consisting of the 62 original Platy-1 subfamilies reported in [1], the two *Aotus* derived subfamilies previously reported in [5], as well as all current *Alu* subfamily consensus sequences obtained from RepBase [9]. This custom library is available as Supplementary File S1. Each RepeatMasker output was analyzed for full-length Platy-1 elements, defined as possessing a start position no more than 4 bp from the 5′ start of the Platy-1 consensus sequence (positions 0 to 4) and an end position of ≥103, and then sorted by the number of insertions per Platy-1 subfamily. These elements along with 500 bp of flanking sequence on both the 5′ (if available) and the 3′ ends of the Platy-1 insertion were used to generate fasta files to compare to the other two Callithrichidae genomes using a locally installed version of BLAT [10]. Platy-1-17 and 17a subfamilies as reported in [1] have consensus sequences only 82 bp long, therefore our full-length filter initially eliminated them from consideration, but they were later included. 

### 2.2. Lineage-Specific vs. Shared Platy-1 Elements

Successive implementations of BLAT [10] were performed for each set of full-length Platy-1 elements including 500 bp flanking sequence, against the other two callithrichid genomes as well as owl monkey (*A. nancymaae*; Anan_2.0), squirrel monkey (*S. boliviensis*; sBol_2.1) and capuchin (*C. imitator*; Cebus imitator_1.0). After each BLAT was completed, the output was searched for shared or unique elements by looking for specific gap sizes between the input Platy-1 sequences and the target genome. Specificity was determined computationally using a custom Python script, “inDepthSpecCheck1_platy-1” to detect an ~85 bp gap compared to other genomes. This program is a modified version of one previously reported for *Alu* element detection (available on link https://github.com/t-beck; accessed on 14 June 2023). Briefly, this program calls an insertion specific if an 85 bp gap is detected in the other comparison genomes (indicating absence of the Platy-1 element) and conversely, determines the element is shared if the gap is not detected. Ambiguous calls were checked again and confirmed by manual inspection of the BLAT alignments in BioEdit [11]. Three specificity groups were investigated in this study, lineage-specific (LS—unique to one tamarin species), *Saguinus*-specific (‘Sag’—shared by both *Saguinus* species but absent from marmoset, *Aotus* and cebids) or callithrichid-specific (‘Call’—shared by the three callithrichids while absent from *Aotus* and cebids). The LS elements from each tamarin genome were computationally extracted to separate fasta files for subfamily characterization. 

### 2.3. COSEG Analysis of Tamarin Platy-1 Subfamilies

A combination of cross_match (http://www.phrap.org/phredphrapconsed.html; accessed on 14 June 2023) and COSEG (www.repeatmasker.org/COSEGDownload.html; accessed on 14 June 2023) analyses were completed to determine if any of the lineage-specific Platy-1 elements extracted from the tamarin genomes represented new subfamilies. These analyses were performed separately for *S. imperator* and *S. midas* to determine if each lineage had independent source nodes of amplification. The analyses were also conducted on the ‘Sag’ set of *Saguinus*-specific insertions to identify any potential new subfamilies that originated prior to the *S. imperator*/*S. midas* split. Platy-1 element sets were aligned to subfamily consensus sequences for Platy-1-6, 1-8 and 1-9 from [1] and exact matches were eliminated. New COSEG derived subfamilies were added to the custom library used for WGS analyses and RepeatMasker [8] was performed again for the three callithrichid genomes, this time using the fasta files containing full-length Platy-1 elements with 500 bp flanking sequence. New subfamilies in which no members were assigned, or in which the new assignment did not improve the respective Smith–Waterman (SW) and percent divergence (% div) scores as compared to the original RepeatMasker run (on WGS), were eliminated. Retained subfamilies were renamed using standardized nomenclature and the new custom library was updated (Supplementary File S2). RepeatMasker was performed again to determine new subfamily assignments and to calculate improved SW and % div scores. 

### 2.4. Neighbor-Joining Tree of Platy-1 Subfamilies

To visualize the placement of the new *Saguinus* derived Platy-1 subfamilies in the context of those previously reported, a Neighbor-Joining tree [12] was generated using MAFFT version 7 [13]. The tree was exported in Newick format and the output was visualized using FigTree v1.4.4. (http://tree.bio.ed.ac.uk/software/figtree/; accessed on 14 June 2023) and exported as a .png file to PowerPoint for annotation. 

## 3. Results

### 3.1. Full-Length Platy-1 Elements

The RepeatMasker output performed on WGS for full-length Platy-1 elements using the original subfamily library are available in Supplementary File S3; Tables S1–S3 and are summarized in Table 1. The numbers for marmoset [calJac4] are comparable to those initially reported for [calJac3] [1]. However, both *S. imperator* and *S. midas* have several thousand Platy-1-8 and 1-9 subfamily members (shown in bold font), resulting in total counts of 13,555 and 11,295 full-length Platy-1 elements, respectively. These are surprisingly large copy numbers, that are five to six times more than that found in *C. jacchus*. The number of full-length elements per subfamily is generally uniform across the three callithrichid genomes between subfamilies Platy-1-1 to 1-7a, with the exception of 1-6g, which appears to have more members in tamarins than in marmoset, and 1-7 and 7a, which appear to have fewer members in tamarins compared to marmoset. Platy-1-17 and 17a subfamilies as reported in [1] have consensus sequences only 82 bp long, therefore our full-length filter initially eliminated them (shown as “0” in Table 1); however, due to their key placement on the subfamily tree, located between Platy-1-8 and 1-9 [1], the total number detected in each genome, regardless of length, is shown in parentheses. However, Platy-1-17 and 17a subfamilies do not have similar intermediate membership values compared to Platy-1-8 and 1-9 and do not appear to be highly active in the *Saguinus* lineage. Of the 62 subfamilies discovered in marmoset, those younger than Platy-1-9a are nearly non-existent in these two tamarin lineages (Table 1) consistent with expectations based on [1].

The total number of full-length Platy-1 elements for each genome and the total number with available flanking sequence (fasta) are listed in Table 2. It is noteworthy that these two values are identical for marmoset [calJac4] and nearly identical for *S. midas* [ASM2_v1], the two chromosome-level genome assemblies. By contrast, the number with available flanking sequence declines considerably for *S. imperator* [SagImp_v1], a scaffold level assembly, presumably due to shorter sequence contigs. Genome assembly statistics are shown in Supplementary File S3; Table S4.

### 3.2. Lineage-Specific vs. Shared Platy-1 Elements

The number of lineage-specific insertions as well as the number of shared elements for ‘Sag’ and ‘Call’ categories calculated from each callithrichid genome are shown in Table 2. We found 7511 full-length Platy-1 elements specific to the *S. imperator* genome and another 8187 unique to the *S. midas* genome. This constitutes the largest expansion of Platy-1 mobile elements reported to date for any platyrrhine lineage, and the most extensive independent radiation of Platy-1 proliferation in any two species from a single genus. The number of shared insertions calculated from each genome are generally consistent, although not a direct match, the differences in genome quality affecting this calculation appear minimal. 

### 3.3. Tamarin Platy-1 Radiation and Pristine A-Tails

**Figure 1:**
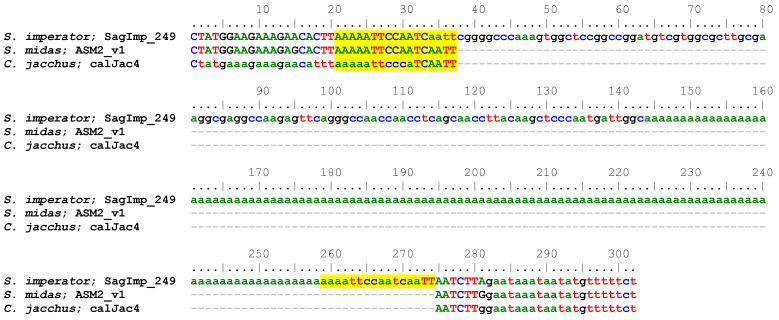
Sequence alignment of an *S. imperator* lineage-specific Platy-1 element with a 119 bp homopolymeric A-tail. The Platy-1 element starts at position 39 (gggg) and extends to position 143, followed by the A-tail. Target site duplications (TSDs) created by the TPRT integration process are shown in yellow highlight. A putative Pol III termination signal (tttt) is located 21 bp downstream of the TSD, which is another potential indicator of mobilization potential as outlined in [1].

Visual inspection of the sequence alignments in BioEdit [11], revealed that many Platy-1 insertions in the *S. imperator* genome possessed very long A-rich tails (A-tails) compared to that previously reported for marmoset [1], in which they were manually counted. Because A-tail length, with no accumulation of other nucleotides in their tails, has been associated with a higher ability to mobilize for *Alu* [14,15,16], we calculated the length of homopolymeric A-tails (no other intervening nucleotides) for each tamarin genome using the Max (Frequency) function in Excel to count the maximum number of consecutive A’s at the 3′ end of each insertion. The length of *S. imperator* pristine A-tails ranged from 1 to 165 bp, including 26 elements that possessed 100 bp or more of consecutive A’s and another 150 insertions with over 50 bp of consecutive A’s (Supplementary File S3; Table S5). An example is shown in Figure 1. This is atypical for retroposons and indicative of very recent integration. The length of *S. midas* pristine A-tails ranged from 2 to 162 bp, but in contrast to *S. imperator*, only three were over 50 bp in length, with only two of these over 100 bp, while the vast majority were <30 bp (Supplementary File S3; Table S5) similar to that previously reported for marmoset [1]. For *S. imperator* LS insertions, there was not the anticipated correlation between longer A-tails and lower percent divergence scores, as all elements seem quite young regardless of A-tail length (Supplementary File S3; Figure S1). These results suggest that both *S. imperator* and *S. midas* likely possess multiple actively mobilizing source elements driving the parallel independent expansion in each lineage.

### 3.4. COSEG Analysis of Tamarin Platy-1 Subfamilies

**Figure 2:**
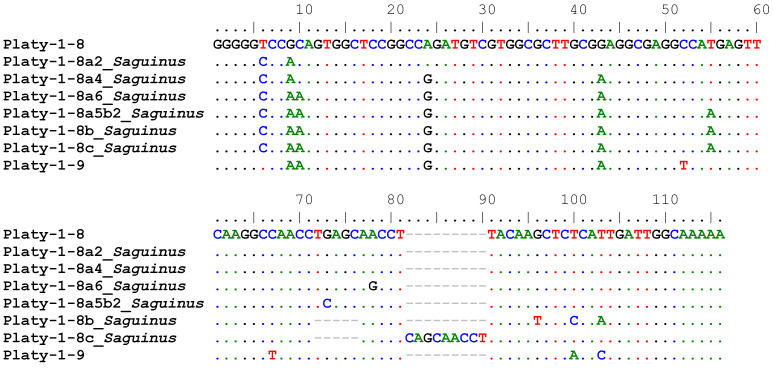
Platy-1 consensus sequence alignment. The consensus sequences of Platy-1-8 and Platy-1-9 were aligned to the newly discovered *Saguinus* subfamilies derived from Platy-1-8 and discovered via COSEG analyses of all full-length Platy-1 elements from the *S. imperator* and *S. midas* genomes. Dots represent a shared nucleotide, dashes represent alignment gaps, while diagnostic substitutions are shown as the corrected base.

COSEG [8] analyses were performed on lineage-specific and *Saguinus*-specific sets of full-length Platy-1 insertions using subfamily consensus sequences for Platy-1-6, 1-8 and 1-9 as reference. Potential new subfamilies derived from Platy-1-6 did not improve RepeatMasker [8] output measures, Smith–Waterman (SW) or percent divergence (% div) scores in any instance and were eliminated. We report six new tamarin Platy-1 subfamilies, aligned in Figure 2, illustrating the accumulation of diagnostic nucleotide changes between Platy-1-8 and 1-9 conventional consensus sequences.

**Figure 3:**
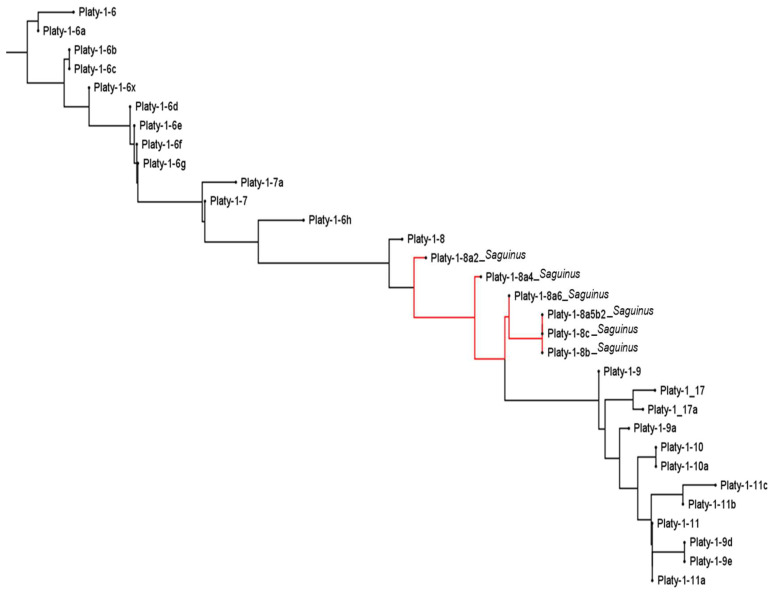
A Neighbor-Joining tree [12] using Platy-1-6 to Platy-1-11 consensus sequences as reported in [1] (black branches) and the six new *Saguinus*-derived subfamilies (red branches). The tree illustrates that Platy-1 radiation in tamarins derived from the Platy-1-8 subfamily lineage. The tree was generated using MAFFT version 7 [13].

The placement of the new *Saguinus* derived Platy-1 subfamilies in the context of those previously reported is shown in Figure 3, as red branches between Platy-1-8 and 1-9. Original subfamilies Platy-1-6 to 1-11 are included on the neighbor-joining tree to span the range of subfamilies detected in *Saguinus* tamarins in this study. The tree is in general agreement with the one reported by Konkel et al. (2016) [1] aside from the placement of the Platy-1-17 and 1-17a subfamilies. Those were manually placed between 1-8 and 1-9 previously, but the current MAFFT [13] analysis places them between 1-9 and 1-9a.

After including these six new Platy-1 subfamilies in the query library, subsequent RepeatMasker analyses resulted in improved accuracy measures. The overall Smith–Waterman score (SW) improved by an average of 208 points (+/− 41) for *S. imperator* and 220 points (+/− 30) for *S. midas* lineage-specific insertions, indicating more precise subfamily assignment. The corresponding percent divergence (% div) from the subfamily consensus sequence was reduced by an average of 6% in both lineages (Supplementary File S3; Tables S6 and S7). The ‘Sag’ group, elements shared by *S. imperator* and *S. midas,* had an improved SW score of 161 points (+/− 70) and a 5% reduction in % div values on average (Supplementary File S3; Tables S8 and S8a), also indicating more accurate subfamily assignment. 

The number of elements from each original subfamily that were reassigned to a new *Saguinus* subfamily is shown in Supplementary File S3 (Tables S6a–8a). Over 90% of the ‘Sag’ group and nearly all members of LS sets were assigned to new COSEG *Saguinus*-derived Platy-1 subfamilies (Table 3). Over 97% of reassigned LS elements were assigned to the youngest new subfamily, Platy-1-8c_*Saguinus* (Table 3; green fill) while only about half of the reassigned ‘Sag’ elements were placed into that youngest subfamily (Table 3; peach fill) and instead have broader representation among subfamilies intermediate in age between Platy-1-8 and Platy-1-8c_*Saguinus* (Table 3). Subfamily Platy-1-8b_*Saguinus*, the immediate precursor to the most dominant subfamily, Platy-1-8c_*Saguinus*, has more members in *S. imperator* than in *S. midas*, but exhibits lower activity in general compared to the other intermediate subfamilies.

Only one element in the ‘Call’ group (ASM2_1321) was unexpectedly assigned to a new subfamily, a single Platy-1-8 element was reassigned to the new 1-8c_*Saguinus* subfamily with improved SW and % div values (Supplementary File S3; Table S8b). However, a more careful review of the sequence alignment for this locus revealed that [calJac4] likely has a near-parallel-insertion rather than an insertion that is identical by descent (Supplementary File S3; Figure S2). All other ‘Call’ group elements retained their original subfamily designation, as expected, providing confidence in the validity of the COSEG derived subfamily structure. 

**Figure 4:**
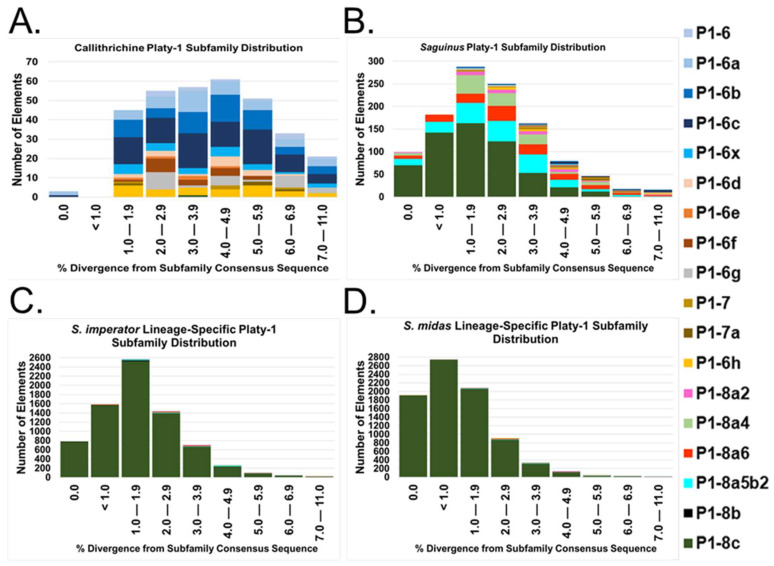
Platy-1 subfamily distribution based on divergence from the consensus sequence. Subfamilies are color-coded and stacked top to bottom by age. (**A**) Platy-1 elements shared in all three callithrichid genomes; (**B**) shared by both *Saguinus* tamarins; (**C**) *S. imperator* lineage specific; (**D**) *S. midas* lineage specific. The divergence from the respective consensus sequence is shown on the x-axes. The y-axes show the number of elements with the indicated divergence.

These results strongly suggest that the expansion of Platy-1 elements in *S. imperator* and *S. midas* initiated after *Saguinus* tamarins diverged from other callithrichids and that it occurred separately from the marmoset-specific Platy-1 radiation reported in [1]. Active founder elements generated new subfamilies with higher mobilization rates leading to the recent proliferation in each lineage. To better evaluate the dynamics of this progression, we calculated the number of Platy-1 insertions based on percent divergence bins (from subfamily consensus sequences) for subfamilies Platy-1-6 to the youngest new subfamily, Platy-1-8c_*Saguinus* (Figure 4). Colors representing each Platy-1 subfamily are layered top to bottom in the same order as dictated by the neighbor-joining tree (Figure 3) and consistent with [1]. The absence of a particular color indicates the absence of that subfamily in the plot. The ‘Call’ subfamily distribution consists largely of Platy1-6-derived and 1-7 subfamilies ranging from light blue at the top to yellow (Platy-1-6h) at the bottom (Figure 4A). ‘Sag’ shared insertions only have small slivers of blue and yellow bands at the top and instead consist primarily of the newly discovered *Saguinus* subfamilies, Platy-1-8a2 (pink) to Platy-1-8c (dark green), with most of the younger insertions (≤ 2% div) belonging to the youngest subfamily (Figure 4B). In sharp contrast, nearly all lineage-specific insertions for *S. imperator* (Figure 4C) and *S. midas* (Figure 4D) belong to the youngest subfamily, Platy-1-8c_*Saguinus*, with *S. midas* having a pronounced leftward shift to the lower percent divergence bins. Using a mutation rate of 0.006024 per base per million years (my) as described previously in [5], 2% divergence corresponds to an age estimate of 3.32 my, while 7% is ~11.62 my. However, Platy-1 elements are only ~100 bp in length, and therefore, each nucleotide corresponds to 1%, or about 1.66 my [1,5], hindering pin-point timing of the observed amplification burst.

In addition to belonging to the youngest subfamily, 66% (4934 of 7511) of *S. imperator* and 82% (6744 of 8187) of *S. midas* lineage-specific Platy-1 elements are <2% diverged from the subfamily consensus sequence, including about 10% (779 of 7511) and 23% (1912 of 8187), respectively, having pristine 0% divergence (Supplementary File S3; Tables S9–S10). About 50% of ‘Sag’ shared insertions are <2% diverged from the subfamily consensus sequence with about 8% pristine (Supplementary File S3; Table S11). These data indicate sharp delineations in Platy-1 subfamily amplification activity during Callithrichidae evolution.

## 4. Discussion

This study shows that *Saguinus* tamarins are experiencing the most robust expansion of Platy-1 MEIs reported to date for any platyrrhine lineage, and the most extensive independent Platy-1 proliferation in any two closely related species. The availability of two separate WGS, for two *Saguinus* species, generated by two separate sources, makes it exceedingly unlikely that these findings are artifactual. Both genomes exhibited nearly the same magnitude of Platy-1 expansion. Also, the fact that the vast majority of Platy-1 element propagation, in both genomes, derives from a single, newly discovered, and (thus far) *Saguinus*-specific subfamily, suggests that other tamarin lineages are likely undergoing similar mobilization processes. In the absence of WGS for other tamarin genomes, the extent to which Platy-1 radiation has impacted other tamarins could be investigated experimentally using PCR, by researchers with access to DNA samples from large numbers of tamarin species and populations, including the Lion tamarins (genus *Leontopithecus*). The youngest and most prolific subfamily, Platy-1-8c_*Saguinus*, contains a distinctive 9 bp insertion in the consensus sequence that could be used to screen short sequence reads. It also has a 5 bp deletion that is shared by Platy-1-8b_*Saguinus*, the immediate precursor subfamily. Compared to the other four intermediate subfamilies derived from Platy-1-8, Platy-1-8b_*Saguinus* has the fewest members, but the consensus sequence clearly places it incremental in diagnostic sequence evolution, prior to the burst of Platy-1-8c_*Saguinus*. Therefore, it is possible that other tamarins, spanning the species tree, could have larger numbers of these subfamily members.

Platy-1 retroposons have now been characterized in six platyrrhine genera, with the bulk of recent activity evident in Callithrichidae. Extensive bursts of MEI activity, such as those observed in tamarins, can dramatically impact genomes and perhaps be deleterious to the host through insertional mutagenesis or post-insertion recombination, similarly to that of the *Alu* family [17,18]. Callithrichids have unique characteristics compared to other platyrrhines, such as diminutive size and twinning [2,19]. Four different genes reportedly have callithrichid-specific nonsynonymous alterations that are possibly associated with these unique features [19]. In marmoset, Platy-1 elements are distributed across all chromosomes, but not evenly; chromosome 4 reportedly has a much lower density than expected; and chromosomes 18, 19 and 22 have a higher density [1]. A comprehensive examination of Platy-1 chromosome distribution and genomic density across lineages is needed, especially in relation to genic regions. Gene annotations are not presently available for tamarin genomes to determine if specific genetic changes that arose during callithrichid evolution relate to the extensive Platy-1 expansion observed in this study. 

Evidence suggests that the expansion of Platy-1 elements in *S. imperator* and *S. midas* initiated after *Saguinus* tamarins branched from other callithrichids and that the marmoset-specific Platy-1 radiation occurred separately. Chromosome painting conducted in *S. midas* showed that tamarins (genera *Saguinus* and *Leontopithecus*) maintain an ancestral callithrichid karyotype, while marmosets (genera *Callithrix* and *Mico*) experienced chromosomal translocations leading to a more derived karyotype [20]. Chromosomal rearrangements could alter the genomic environment of potential source elements, inactivating some while enhancing the mobility of others. The discovery that both *S. imperator* and *S. midas* possess nearly six times more full-length Platy-1 elements than *C. jacchus*, and that over 7500 Platy-1 insertions have integrated independently in each tamarin lineage since their divergence, suggests that tamarins harbor different, and more active, source elements than marmosets, generating rapid proliferation of Platy-1 elements over a relatively short evolutionary time frame. 

The initial characterization of Platy-1 elements in marmoset suggested that their mobilization rate increased sharply with the rise of the marmoset common ancestor [1], a dynamic consistent with the stealth model of amplification in which a small number of active source elements propagate at a very low rate, perhaps over millions of years, before some daughter elements become highly active and generate many insertions relatively quickly [21]. The results of this study strongly support the assertion that Platy-1 propagation increased rapidly coinciding with the emergence of callithrichids, producing daughter copies from multiple subfamilies in parallel. Multiple Platy-1 subfamilies derived from 1-6 and 1-7 were active in parallel and are shared by the three callithrichids, and then, a rapid burst of Platy-1-8 derived subfamilies occurred in tamarins, all of which produced multiple progeny elements. However, without the discovery of this sudden offshoot of Platy-1-8 radiation in tamarins, the observed subfamily distribution is nearly identical to that obtained from PCR analyses in marmoset in which subfamilies younger than Platy-1-9 were restricted to marmosets [1,5] (Storer et al. (2019) Supplementary File S3; Figure S2). Therefore, it is important to determine if *Saguinus*, and tamarins in general, are experiencing a unique amplification dynamic, or to what extent other platyrrhine lineages, including other marmosets, may have experienced similar independent bursts of amplification. There appears to be rather sharp delineations in Platy-1 subfamily amplification at different time points in callithrichid evolution that could have influenced phylogeny and speciation. Nearly dormant source elements may have persisted among members of family Pitheciidae or Atelidae that have since undergone independent radiation, after the split that led to the three-family clade of Cebidae, Aotidae and Callithrichidae. Such potential offshoots might appear similar to the moderate expansion of Platy-1-4b and 4b3 elements detected in *Aotus* [5] or could be much more dramatic. 

Platy-1 elements mobilize via TPRT and therefore compete for the same LINE (L1) enzymatic machinery that *Alu* elements do to achieve successful propagation. *Alu* content has not yet been characterized in tamarins specifically, to determine if *Alu* amplification rates are lower due to the vast expansion of Platy-1 elements, as compared to other reported platyrrhine genomes [22,23,24,25,26]. However, we have observed that Platy-1 elements in tamarins and other genomes [5] are often flanked by one or more *Alu* elements. Also, the L1 lineage is actively evolving in the *Saguinus* genus, generating lineage-specific subfamilies [27]. Therefore, the functional mechanisms for TPRT should theoretically be unrestrained in *Saguinus*, to the extent that MEI genomic density can evade host defenses that slow retrotransposition. These factors could mean that the sudden burst of Platy-1-8 expansion in tamarins is unique in this regard, perhaps fueled by their rapid radiation with reticulated evolution [28]. The extent to which the genomic density and, hence, the availability of these amplification mechanisms impacts *Saguinus* and other platyrrhine genomes, perhaps differently, should be explored. Including all available platyrrhine genomes in the next study will help address these issues.

Finally, a focus of this study was the identification of recently integrated, or young insertions. It is important to note that the use of ≤2% divergence from a respective consensus sequence, as a RepeatMasker output metric for being considered “young” is historically based on *Alu* element amplification dynamics [14,29]. A 300 bp *Alu* element with 2% divergence translates to about six random mutations due to age related decay. A Platy-1 element is only about 100 bp long, and therefore, 2% divergence is only two mutations, whereas 6% (six mutations) would be equivalent using the *Alu* guidelines. The tamarin Platy-1 elements in this study might be much more recent than a 2% divergence measurement implies. Future studies should take this factor into consideration, while also assessing levels of insertion polymorphism.

## 5. Conclusions

At nearly six times that of *C. jacchus*, *S. imperator* and *S. midas* tamarins exhibit the most extensive expansion of Platy-1 retroposons reported to date and the highest proliferation rate in independent species from a single genus. Six new *Saguinus*-specific subfamilies are reported that derived from Platy-1-8 with the primary burst of current activity occurring in the youngest subfamily, Platy-1-8c_*Saguinus*. Future work involves analyzing other currently available platyrrhine WGS genomes for Platy-1 content and genomic distribution to determine if tamarins have experienced unique evolutionary forces related to Platy-1 mobilization dynamics.

## Figures and Tables

**Table 1:** Platy-1 subfamily distribution for three callithrichid genomes.

**Subfamily**	**P1-1**	**P1-2**	**P1-2a**	**P1-2b**	**P1-3**	**P1-5**	**P1-4**	**P1-4a**	**P1-4b**	**P1-4b3**	**P1-6**	**P1-6a**	**P1-6b**
calJac4	60	23	51	39	25	18	18	19	2	1	14	67	76
SagImp_v1	56	27	53	45	22	43	22	20	2	0	11	70	77
ASM2_v1	63	19	59	37	21	20	18	20	1	0	16	65	80
**Subfamily**	**P1-6c**	**P1-6x**	**P1-6d**	**P1-6e**	**P1-6f**	**P1-6g**	**P1-7**	**P1-7a**	**P1-6h**	**P1-8**	**P1-17**	**P1-17a**	**P1-9**
calJac4	117	32	13	16	27	26	59	19	51	28	0 (7)	0 (1)	36
SagImp_v1	118	34	19	16	29	101	29	10	59	**6824**	0 (11)	0 (137)	**5818**
ASM2_v1	127	34	21	10	29	40	28	10	58	**5778**	0 (2)	0 (4)	**4684**
**Subfamily**	**P1-9a**	**P1-10**	**P1-10a**	**P1-9b**	**P1-9c**	**P1-9d**	**P1-9e**	**P1-11**	**P1-11a**	**P1-11b**	**P1-11c**	**P1-12**	**P1-12a**
calJac4	28	41	16	99	47	34	12	33	16	20	35	19	11
SagImp_v1	36	1	0	0	1	0	0	0	0	0	0	0	1
ASM2_v1	44	5	1	1	0	0	0	0	0	2	0	0	0
**Subfamily**	**P1-12b**	**P1-12d**	**P1-12e**	**P1-12f**	**P1-12c**	**P1-13f**	**P1-13**	**P1-13e**	**P1-15a**	**P1-13c**	**P1-14a**	**P1-14**	**P1-14b**
calJac4	71	21	19	19	22	55	4	2	93	53	0	0	6
SagImp_v1	0	0	0	0	0	0	0	0	0	0	0	0	0
ASM2_v1	0	0	0	0	0	0	0	0	0	0	0	0	1
**Subfamily**	**P1-15**	**P1-13g**	**P1-16**	**P1-16a**	**P1-16b**	**P1-16c**	**P1-16d**	**P1-16e**	**P1-16f**	**P1-13b**	**P1-13a**	**P1-13d**	**Total**
calJac4	279	50	39	19	23	9	10	9	91	18	33	38	2231
SagImp_v1	0	0	1	0	0	0	0	9	1	0	0	0	13,555
ASM2_v1	1	0	1	0	0	0	0	1	0	0	0	0	11,295

The number of subfamily specific full-length Platy-1 elements detected by RepeatMasker in marmoset [calJac4], *S. imperator* [SagImp_v1] and *S. midas* [ASM2_v1]. Bold font indicates the number of Platy-1-8 and 1-9 subfamily members found in each tamarin genome. Numbers in parentheses indicate the total number Platy-1-17 and 17a members detected in each genome regardless of length.

**Table 2:** Number of Platy-1 elements by Genome and Specificity.

Genome	calJac4	SagImp_v1	ASM2_v1
Total Full-length (FL)	2231	13,555	11,295
FL w/fasta sequence	2231	10,581	11,294
Lineage-Specific (LS)	1452	7511	8187
*Saguinus*-specific (Sag)	n/a	1169	1149
Callithrichidae (Call)	323	325	340

**Table 3:** Number of lineage-specific (LS) and *Saguinus* (Sag) Platy-1 elements assigned to new COSEG subfamilies.

	*S. imperator*				*S. midas*			
COSEG subfamilies (sf)	LS	% of 7511	Sag	% of 1169	LS	% of 8187	Sag	% of 1149
Platy-1-8a2_*Saguinus*	6	0.08	39	3.34	1	0.01	36	3.13
Platy-1-8a4_*Saguinus*	19	0.25	94	8.04	15	0.18	109	9.49
Platy-1-8a6_*Saguinus*	63	0.84	122	10.44	62	0.76	127	11.05
Platy-1-8a5b2_*Saguinus*	50	0.67	194	16.60	42	0.51	195	16.97
Platy-1-8b_*Saguinus*	29	0.39	1	0.09	2	0.02	0	0.00
Platy-1-8c_*Saguinus*	7298	97.16	623	53.29	8053	98.36	587	51.09
Total assigned to new sf	7465	99.39	1073	91.79	8175	99.85	1054	91.73

## Data Availability

The algorithms used in this study are available on GitHub (https://github.com/t-beck; accessed on 14 June 2023). The Supplementary data files are available on the online version of this paper and through the Batzer Lab website under publications, https://biosci-batzerlab.biology.lsu.edu/; accessed on 14 June 2023.

## Supplementary Materials

The following supporting information can be downloaded at: https://www.mdpi.com/article/10.3390/genes14071436/s1, Supplementary File S1 is a custom RepeatMasker library consisting of 64 Platy-1 subfamily consensus sequences [1,5] and conventional *Alu* sequences [9] used for WGS analysis in this study. Supplementary File S2 is an amended RepeatMasker library including the six new *Saguinus* Platy-1 subfamilies discovered in this study. Supplementary File S3 is an Excel file containing supplementary Figures S1 and S2 and Tables S1–S11.
